# Identification of the Molecular Mechanism by which TLR Ligation and IFN-γ Synergize to Induce Mig

**DOI:** 10.1080/10446670410001670535

**Published:** 2004-03

**Authors:** Jonathan D. Powell, Sada Boodoo, Maureen R. Horton

**Affiliations:** 1 Department of Oncology Johns Hopkins University School of Medicine 1650 Orleans Street, Room 443 Baltimore MD 21231 USA; 2 Department of Medicine Johns Hopkins University School of Medicine 1830 East Monument, Street Suite 500 Baltimore MD 21205 USA

## Abstract

Monokine Induced by Interferon- (MIG), a CXC chemokine, is a potent inducer of T-cell chemotaxis and activation and has been implicated in the host response to viral infections and tumor immunity as well as in the pathogenesis of autoimmunity and transplant rejection. Although it is known that the Toll-Like Receptor-4 (TLR-4) ligand LPS synergizes with IFN-γ to induce MIG expression in macrophages, the molecular mechanisms responsible for the synergy have yet to be elucidated. We determined that the marked synergy between LPS and IFN-γ on MIG mRNA expression in mouse macrophages is a result of LPS-induced NF-_κ_B and IFN-γ-induced STAT. The synergy was not dependent on new protein synthesis, was independent of TNF-α, and occurred at the level of gene transcription. We identified 2 NF-_κ_B sites located at -154 and -129 of the MIG promoter proximal to the -responsive element that mediated this effect. Finally, we demonstrated that other TLR ligands (zymosan, double stranded RNA and CpG) synergized with IFN-γ to induce MIG in an NF-_κ_B dependent fashion. These data emphasize the ability of bacterial and viral products to activate/modify immune responses and promote adaptive T cell immunity through the NF-_κ_B pathway.


*Clinical & Developmental Immunology*, March 2004, Vol. 11 (1), pp. 77–85

Taylor & Francis healthsciences

### Identification of the Molecular Mechanism by which TLR Ligation and IFN-γ Synergize to Induce Mig

JONATHAN D. POWELL[a], SADA BOODOO[b] and MAUREEN R. HORTON[b,*]

[a]*Department of Oncology, Johns Hopkins University School of Medicine, 1650 Orleans Street, Room 443, Baltimore, MD 21231, USA;* [b]*Department of Medicine, Johns Hopkins University School of Medicine, 1830 East Monument, Street Suite 500, Baltimore, MD 21205, USA*

Monokine Induced by Interferon-γ (MIG), a CXC chemokine, is a potent inducer of T-cell chemotaxis and activation and has been implicated in the host response to viral infections and tumor immunity as well as in the pathogenesis of autoimmunity and transplant rejection. Although it is known that the Toll-Like Receptor-4 (TLR-4) ligand LPS synergizes with IFN-γ to induce MIG expression in macrophages, the molecular mechanisms responsible for the synergy have yet to be elucidated. We determined that the marked synergy between LPS and IFN-γ on MIG mRNA expression in mouse macrophages is a result of LPS-induced NF-κB and IFN-γ-induced STAT. The synergy was not dependent on new protein synthesis, was independent of TNF-α, and occurred at the level of gene transcription. We identified 2 NF-κB sites located at − 154 and − 129 of the MIG promoter proximal to the γ-responsive element that mediated this effect. Finally, we demonstrated that other TLR ligands (zymosan, double stranded RNA and CpG) synergized with IFN-γ to induce MIG in an NF-κB dependent fashion. These data emphasize the ability of bacterial and viral products to activate/modify immune responses and promote adaptive T cell immunity through the NF-κB pathway.

*Keywords*: Adaptive immunity; Innate immunity; Interferon-γ; MIG; NF-κB; TLR

### INTRODUCTION

The Toll-Like receptors (TLRs) play a critical role in the induction of the innate immune response. These receptors have evolved to recognize pathogen associated molecular patterns that are integral components of lipopolysaccharides, zymosan, flagellin, unmethylated CpG and double stranded RNA (dsRNA) (Takeuchi and Akira, 2001; Dunne and O'Neill, 2003). In this fashion, the host can rapidly respond to infections by elaborating cytokines, chemokines and inflammatory enzymes as well as reactive oxygen species. TLR engagement also activates macrophages and dendritic cells, increasing their cell surface expression of co-stimulatory and adhesion molecules (Jones *et al.*, 2001; Dunne and O'Neill, 2003). Thus TLR ligation heralds infection (danger) and induces the necessary second signals for adaptive T cell immunity.

Activated macrophages play an essential role in inflammation by releasing of a variety of mediators including reactive oxygen and nitrogen species, proteases, chemokines, cytokines and growth factors (Nathan, 1987). IFN-γ modulates macrophage effector functions and regulates the inflammatory response of organisms to host insults such as endotoxin by enhancing macrophage microbiocidal and tumoricidal activity as well as specific chemokine production (Luster *et al.*, 1985; Maeyer and Maeyer-Guignard, 1992). LPS, the structural component of gram-negative bacteria, is one of the most potent microbial activators of inflammation and inducers of macrophage cytokines and chemokines. Together IFN-γ and LPS can synergizes to further potentate the production of endogenous mediators of inflammation (Farber, 1992,1993; Gasperini *et al.*, 1999).

IFN-γ induces macrophage expression of the chemokine Monokine Induced by interferon-γ (MIG) (Farber, 1990,1992,1993; Liao *et al.*, 1995; Gasperini *et al.*, 1999). This chemokine is up-regulated in chronic inflammation as well as in viral and protozoan infections and its expression correlates with IFN-γ expression (Amichay *et al.*, 1996). In addition to its roles in T-cell trafficking, chemotaxis and activation, MIG is one of the CXC chemokines with angiostatic properties (Liao *et al.*, 1995; Strieter *et al.*, 1995; Loetscher *et al.*, 1996). Thus, MIG may play an important role in regulating tissue granulation and remodeling as well as slowing down tumor growth by inhibiting angiogenesis. To date, IFN-γ alone has been identified as an inducer of MIG in macrophages (Farber, 1990). While several investigators have described the synergistic enhancement of IFN-γ induced MIG expression by LPS in macrophages,

*Corresponding author. Tel.: + 1-410-955-3467. Fax: + 1-410-955-0299. E-mail: mhorton2@jhem.jhmi.edu

ISSN 1740-2522 print/ISSN 1740-2530 online © 2004 Taylor & Francis Ltd
DOI: 10.1080/10446670410001670535

the molecular mechanisms mediating this effect are unknown (Farber, 1990; Gasperini *et al.*, 1999).

In this report we demonstrate that the maximal production of MIG by macrophages is the result of the collaboration between Toll-like receptor engagement and IFN-γ. We define the molecular mechanisms responsible for the striking synergy by which LPS-induced NF-κB (TLR4 ligation), acting in conjunction with IFN-γ, regulates the immune response by influencing the expression of specific chemokines. In addition, we extend our findings to other TLR ligands showing that they too can synergize with IFN-γ, in an NF-κB dependent fashion, to enhance MIG production.

### MATERIAL AND METHODS

### Cells

MH-S cells, a mouse alveolar macrophage cell line, were purchased from American Type Culture Collection, Rockville, MD. (Mbawuike and Herscowitz, 1989). Cells were maintained in RPMI 1640 supplemented with 10% heat-inactivated low-LPS fetal bovine serum, 1% penicillin-streptomycin and 1% glutamine (Biofluids, Rockville, MD) at 37°C under 5% $CO_2$. Thioglycollate-elicited peritoneal macrophages were obtained from TNF-α null, p50 NF-κB null and control mice (Jackson, Bar Harbor, ME) as previously described (Horton *et al.*, 1998).

### Chemicals and Reagents

Recombinant mouse IFN-γ (specific activity, $3.0 \times 10^5$ U/ml with endotoxin level less than 0.2 ng/mg) was purchased from Invitrogen. Proteosome inhibitor-1, Wortmannin and MEK inhibitor PD98059 were purchased from Calbiochem, LaJolla, Ca. Cycloheximide, LPS (pseudomonas aeroginosa serotype 10), zymosan and dsRNA were purchased from Sigma. CpG was synthesized by Oligos Etc. Inc.

### Northern Analysis of mRNA Production

RNA was extracted from confluent cell monolayers of MH-S cells via Trizol reagent (Invitrogen) or from peritoneal macrophages with RNeasy (Qiagen) per manufacturer's guidelines. Northern blot analysis was performed as described previously (Horton *et al.*, 1998).

### Preparation of Nuclear Extracts and Electrophoretic Mobility Shift Assays

Nuclear extracts from MH-S cells were prepared as previously described (Horton *et al.*, 2002; Powell *et al.*, 1999). Electrophoretic mobility shift assays (EMSA) were conducted using 6% polyacrylamide gels, as previously described (Horton *et al.*, 2002). Nuclear extracts (5 μg) were incubated at RT for 30 min with 30,000 cpm of $^{32}P$ end-labeled, double-stranded oligonucleotide probe (10–50 pg), 0.1–1 μg of denatured salmon sperm DNA (Invitrogen) and buffer prior to gel electrophoresis at 4°C. The following dsDNA probes were used: −154 site gcagaaattccctgggatctgag, −154 M mutant gcagaaatt-cAAtgggatctgag, −129 site tagggttttccccaggacgatc and −129 M mutant tagggttttcAAcaggacgatc. Additionally, dsDNA probes of the following consensus sequences were used for cold competition analysis: NF-κB, tagggttttccc-caggacgatc and STAT-1α, catgttatgcatattcctgtaagtg (Santa Cruz Biotechnology). For supershift analysis, nuclear extracts were simultaneously incubated with 1 μg of the indicated antibody and the labeled probe prior to EMSA. The following antibodies were obtained from Santa Cruz Biotechnology: STAT-1 p84/p91, NF-κB p50, NF-κB p65 and NF-κB p52.

### RNA and EMSA Analysis

Blots were developed on a STORM PhosphorImager (Molecular Dynamics, Sunnyvale, CA). Quantification of bands was determined by the PhosphorImager using a fixed area with the object average program for determining the background (ImageQuant; Molecular Dynamics) to account for inter-lane background variation.

### Transient Transfections

Transient transfections of the MH-S cells with wild type and mutated MIG promoter reporter constructs were performed as previously described using lipofectamine 2000 per manufacture guidelines (Invitrogen) (Horton *et al.*, 2002). Luciferase expression was measured using a Dual Luciferase Kit (Promega) and a Zylux femtomaster FB-12 luminometer.

### Statistical Analysis

Statistical analysis was performed between groups using an ANOVA factorial analysis program from Statview (Abacus Concepts). A difference between groups of $p < 0.05$ was considered significant.

### RESULTS

### TLR Ligands Synergize with IFN-γ to Induce MIG mRNA Expression in Mouse Macrophages

Previously it has been reported that the TLR-4 ligand LPS can synergize with IFN-γ to induce MIG expression by an unknown mechanism (Farber, 1992; Gasperini *et al.*, 1999; Sun *et al.*, 2001). We further investigated the role of other TLR ligands in inducing MIG expression with and without IFN-γ in macrophages. The alveolar macrophage cell line, MH-S, was simultaneously stimulated with the TLR ligands: zymosan 10 μg/ml (TLR-2), dsRNA 50 ug/ml (TLR-3), LPS 10 ng/ml (TLR-4) and

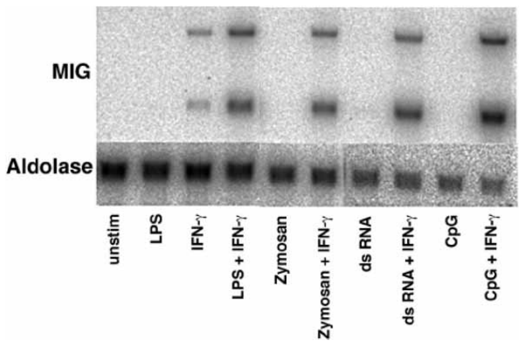

FIGURE 1 TLR ligands and IFN-γ synergistically induce MIG mRNA expression in macrophages. Northern analysis of mRNA from MH-S macrophages stimulated with LPS (10 ng/ml), zymosan (10 μg/ml), dsRNA (50 μg/ml) or CpG (10 μg/ml) +/− IFN-γ (300 U/ml) for 6 h. These data are representative of three experiments.

unmethylated CpG 10 μg/ml (TLR-9) +/− IFN-γ (300 U/ml) for 6 h, total RNA was isolated and Northern analysis performed (Fig. 1). Whereas IFN-γ alone resulted in minimal induction of MIG (note the typical MIG mRNA doublet), none of the TLR ligands alone induced MIG. In striking contrast however, there was marked synergy between each of the TLR ligands tested and IFN-γ on the induction of MIG RNA. Similar results were observed with peritoneal derived and bone marrow derived primary macrophages (data not shown). Thus, although TLR ligation in and of itself does not promote MIG expression, in collaboration with IFN-γ it markedly enhances MIG production.

### The TLR-4 Ligand LPS Synergizes with IFN-γ to Induce MIG mRNA Expression in Mouse Macrophages in a Time and Dose Dependent Fashion

We further investigated the molecular mechanisms mediating the synergy between the TLR-4 ligand LPS and IFN-γ on MIG expression by mouse macrophages. MH-S alveolar macrophages were simultaneously stimulated with LPS (10 ng/ml) and IFN-γ (300 U/ml) for varying time intervals and total RNA was harvested for Northern analysis. The synergy between LPS and IFN-γ on MIG expression was seen as early as 3 h after simultaneous stimulation, peaked after 6–9 h and decreased towards baseline after 24 h of stimulation (data not shown). Additionally, as expected, LPS failed to independently induce and IFN-γ only minimally induced MIG mRNA expression at any time point.

We also determined the dose-response relationships for HA and IFN-γ induced MIG expression in macrophages. Northern analysis for MIG was performed on total RNA from MH-S macrophages stimulated with varying concentrations of LPS with a constant concentration of IFN-γ (Fig. 2A). The synergy between LPS and IFN-γ on MIG gene expression occurred with as little as 0.1 ng/ml LPS and was maximal at 1 μg/ml LPS in the presence of 300 U/ml IFN-γ. The converse experiment was performed using a constant concentration of LPS (10 ng/ml) and varying the concentration of IFN-γ (Fig. 2B). The synergy between LPS and IFN-γ occurred with as little as 1 U/ml IFN-γ and was maximal at 1000 U/ml IFN-γ. Importantly, at lower doses of IFN-γ, which alone did not induce detectable MIG, LPS was able to markedly upregulate MIG expression.

### Cycloheximide does not Inhibit the Synergy between the TLR-4 Ligand LPS and IFN-γ on MIG Expression in Mouse Macrophages

In order to dissect the molecular mechanisms mediating this synergy, we examined the potential role of new protein synthesis on MIG mRNA expression to determine if TLR ligation was able to synergize with IFN-γ to induce MIG without an intermediate protein. MH-S macrophages were pretreated with the protein synthesis inhibitor

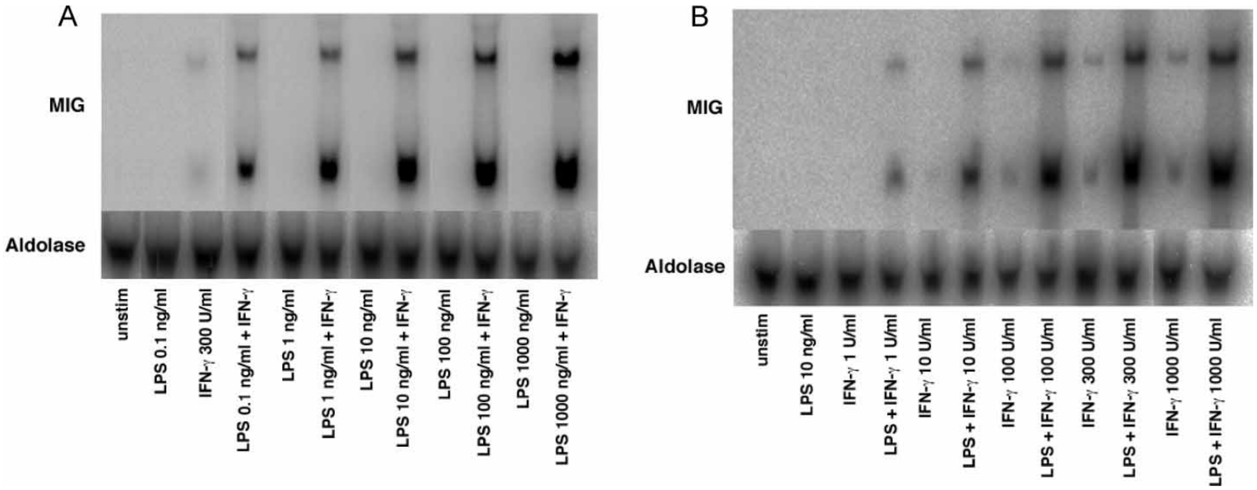

FIGURE 2 Dose response of LPS and IFN-γ for MIG gene expression in macrophages. (A) Northern analysis of mRNA from MH-S macrophages stimulated with increasing doses of LPS +/− IFN-γ (300 U/ml) for 6 h. (B) Northern analysis of mRNA derived from MH-S cells stimulated with increasing doses IFN-γ +/− LPS (10 ng/ml) for 6 h. These data are representative of four experiments.

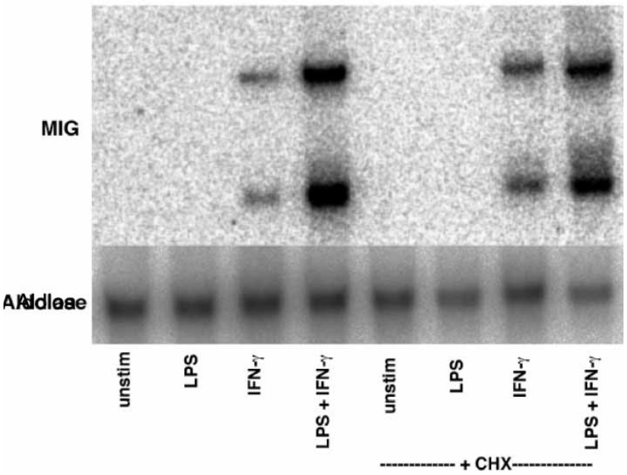

FIGURE 3 Effect of CHX on the synergy between HA and IFN-γ on MIG expression. MH-S macrophages were pretreated with CHX (10 μg/ml) for 30 min before and during stimulation +/− LPS (10 ng/ml) and +/− IFN-γ (300 U/ml) for 6 h. mRNA was isolated and Northern analysis performed. These data are representative of four identical experiments.

cycloheximide 10 μg/ml (CHX) for 30 min before the addition of LPS (10 ng/ml) and IFN-γ (300 U/ml) for 6 h. As seen in Fig. 3, CHX did not significantly inhibit MIG production by IFN-γ or LPS + IFN-γ stimulated macrophages (Fig. 3). Thus, the synergistic induction of MIG gene expression by LPS and IFN-γ does not require new protein synthesis.

### The Synergy between the TLR-4 Ligand LPS and IFN-γ on MIG Gene Expression is Independent of TNF-α

It has been previously published that TNF-α and IFN-γ synergize to induce MIG mRNA in fibroblasts (Ohmori and Hamliton, 1995; Ohmori *et al.*, 1997). Additionally, it is well known that LPS can induce TNF-α expression by mouse macrophages (Hiroi and Ohmori, 2003; Ohmori and Hamliton, 1995; Ohmori and Hamilton, 1993). While our data demonstrating that the synergy induced by TLR ligation does not require new protein synthesis suggests that TNF-α is not involved in enhancing MIG expression, we further investigated the potential role of TNF-α in the LPS and IFN-γ induced synergy of MIG. We isolated total RNA from LPS and IFN-γ stimulated thioglycollate elicited peritoneal macrophages from TNF-α null mice as well as littermate controls (Marino *et al.*, 1997). As shown in Fig. 4, there was no difference in the synergistic induction of MIG by LPS and IFN-γ despite the complete absence of TNF-α. Thus, unlike what has been shown in fibroblasts, the synergy between LPS and IFN-γ on MIG gene expression in macrophages is independent of TNF-α expression. These data support the concept that in macrophages direct TLR and IFN-γ signaling accounts for the synergy.

### The Synergy between TLR Ligation and IFN-γ on MIG Expression Requires NF-κB

In order to determine the signaling pathways mediating the synergy between TLR ligation and IFN-γ on MIG expression, we isolated total mRNA from MH-S macrophages stimulated with the TLR-4 ligand LPS +/− IFN-γ in the presence of various inhibitors of signal transduction. LPS is known to activate the MAP-kinase, Protein Kinase C and NF-κB pathways. The synergy between the LPS + IFN-γ on MIG expression was inhibited by proteosome-1 10 μg/ml (PS-1) (an inhibitor of the NF-κB activation pathway) but not by rottlerin 3 μg/ml (a protein kinase c inhibitor), Wortmannin 1 μg/ml (an inhibitor of PI-3 Kinase) or the MEK inhibitor PD98059 2.5 μg/ml (Fig. 5A). Interestingly, we found that the induction of MIG by IFN-γ alone was not inhibited by PS-1 (Fig. 5B), suggesting that IFN-γ-induced MIG was not dependent on NF-κB activation. Additionally, similar

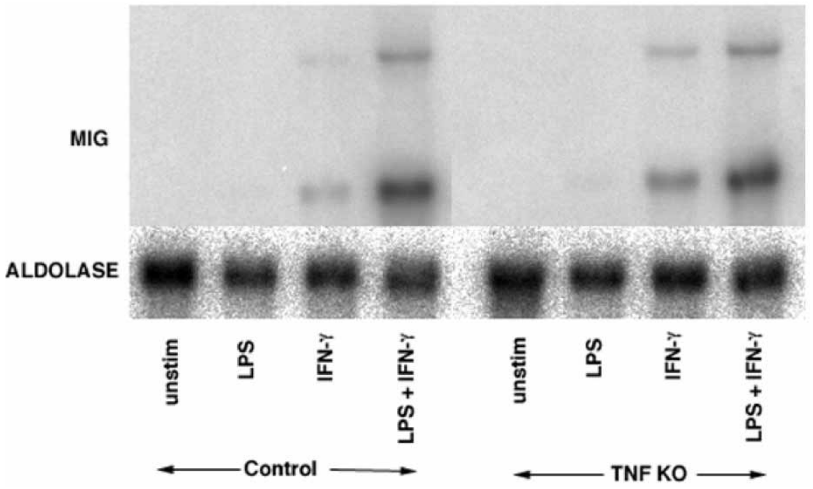

FIGURE 4 TNF-α is not required for the synergistic induction of MIG by LPS and IFN-γ in primary peritoneal macrophages. mRNA was isolated and Northern analysis was performed on thioglycollate elicited peritoneal macrophages from TNF −/− and control C57BL6 mice stimulated with LPS (10 ng/ml) +/− IFN-γ (300 U/ml) for 6 h. This is a representative blot of three identical experiments.

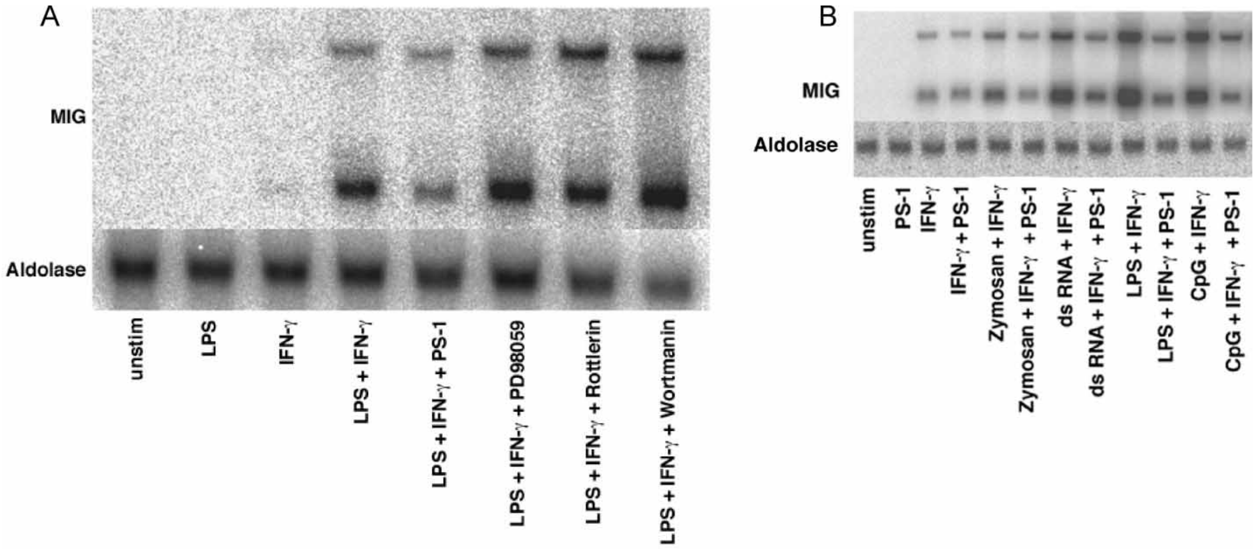

FIGURE 5 The synergy between TLR ligands and IFN-γ on MIG expression requires NF-κB. MH-S macrophages were stimulated with LPS (10 ng/ml) +/− IFN-γ (300 U/ml) for 6 h in the presence or absence of the indicated inhibitors: PS-1 10 μg/ml, PD98059 2.5 μg/ml, rottlerin 1 μg/ml and wortmannin 3 μg/ml (A) or TLR ligands (zymosan 10 μg/ml, dsRNA 50 μg/ml, LPS 10 ng/ml or CpG 10 μg/ml) + IFN-γ (300 U/ml) + PS-1 (10 μg/ml) for 6 h (B). RNA was isolated and Northern analysis was performed. This experiment is representative of three identical experiments.

experiments were performed with various other TLR ligands confirming the need for NF-κB in mediating the synergy (Fig. 5B). Thus the data in Fig. 5 suggests that TLR ligation-induced NF-κB activity plays a role in the synergy between TLR ligands and IFN-γ on MIG transcription. Of note, the cell viability was equivalent in cultures with and without the inhibitors.

### NF-κB p50/p50 Homodimers and p50/p65 Heterodimers Bind to the −154 and −129 NFκB-like Sites on the 5′ MIG Promoter

Given that there are the two NF-κB binding sites at −154 and −129 of the MIG promoter, we wanted to determine if TLR signaling utilized these MIG promoter sites. First we performed EMSAs of nuclear extracts from MH-S macrophages stimulated with LPS +/− IFN-γ for 1 h. Figure 6A demonstrates LPS induced binding of a complex to the −154 site that was competed away by unlabeled consensus NF-κB probe but not unlabeled consensus STAT-1α (note NF-κB appearing as two bands). In addition, supershifts with NF-κB p50 and p65 antibodies show that the proteins binding to the −154 site consisted of both p50/p50 homodimers and p50/p65 heterodimers. The anti-p50 antibody supershifts both bands while the anti-p65 antibody only supershifts the lower band. As a control, anti-p52 antibody failed to supershift either band. Furthermore, this NF-κB binding was specific in that no binding was observed in EMSAs using a probe containing a 2 bp mutation at the −154 site (−154 M) (Fig. 6A).

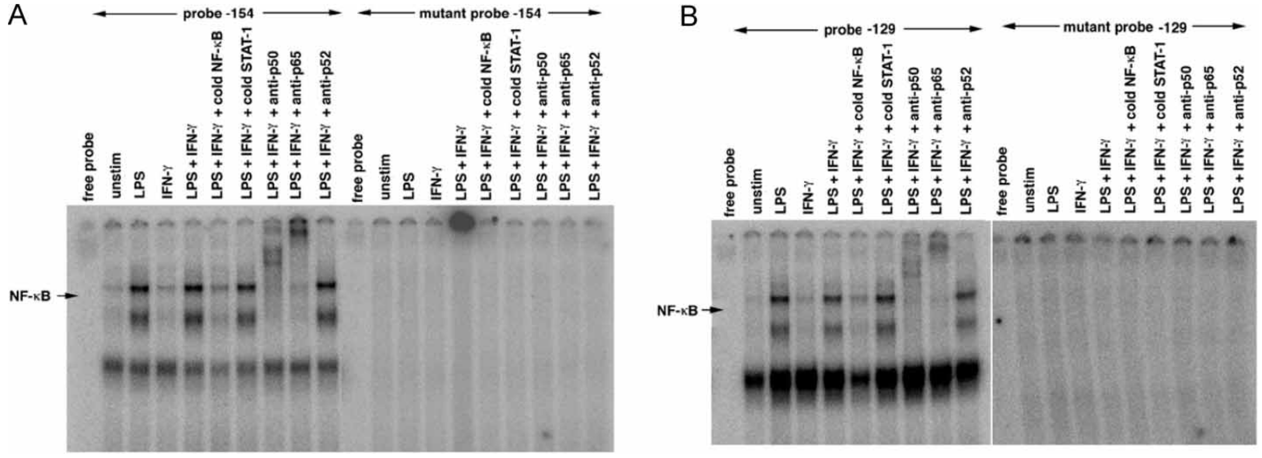

FIGURE 6 NF-κB p50/p50 homodimers and p50/p65 heterodimers bind to the −154 and −129 NFκB-like sites on the 5′ MIG promoter. EMSA was performed using nuclear extracts from MH-S macrophages stimulated with LPS (10 ng/ml) +/− IFN-γ (300 U/ml) for 1 h and radiolabeled DNA probes from the MIG promoter containing the −154 (A) or −129 (B) NF-κB-like site or a mutant −154 site (A) or mutant −129 (B) sites. Competition was performed with unlabeled probes consisting of consensus NF-κB or STAT-1α and supershifts with the indicated antibodies. This experiment is representative of four identical experiments.

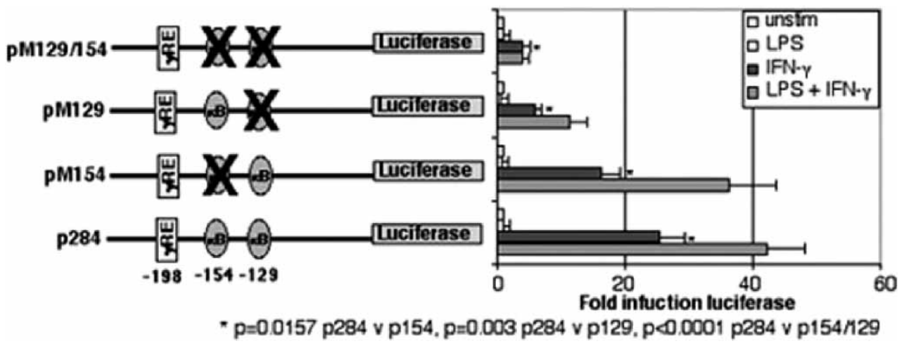

FIGURE 7 The − 154 and − 129 NF-κB-like sites are necessary for synergy between LPS and IFN γ as well as the induction of MIG by IFN-γ alone. MH-S macrophages were transfected with constructs containing 284 bp of the 5′ MIG promoter +/− point mutations in the − 154 (pM154), the − 129 (pM129) or the − 154 + − 129 (pM154/129) sites upstream of a luciferase reporter. Transfected cells were stimulated with LPS (10 ng/ml) +/− IFN-γ (300 U/ml) for 18 h. Promoter activity was assayed by luciferase activity. These data are the result of four identical experiments.

Similar data were obtained from EMSAs performed using a 22 bp DNA probe containing the − 129 site from the MIG promoter and nuclear extracts from MH-S macrophages stimulated with LPS +/− IFN-γ for 1 h. Using the − 129 probe, LPS induced the up-regulation of a protein-DNA complex that was competed away by unlabeled consensus NF-κB but not unlabeled consensus STAT-1α probes. This complex was also supershifted by antibodies to p50 and p65 but not STAT-1α indicating the presence of both p50/p50 and p50/p65 dimers (Fig. 6B). Furthermore, the specificity of NF-κB binding to this site was confirmed by the inability of -129 M probe (identical probe except for a 2 bp mutation at the predicted NF-κB binding site) to bind the protein complex (Fig. 6B). Interestingly, there is also a faint band running at the same level as the NF-κB using either the − 129 and − 154 probes in the unstimulated and IFN-γ alone stimulated conditions (Figs. 6A and B) that is competed with unlabeled consensus NF-κB (data not shown). These data along with our previously published results, suggest that even in resting or IFN-γ alone treated cells, NF-κB family members are constitutively expressed (Horton *et al.*, 2002).

### Both the − 154 and − 129 NF-κB-like Sites on the MIG Promoter are Necessary for the Synergistic Induction of MIG Expression by LPS + IFN-γ as well as for Maximal Induction of MIG by IFN-γ Alone

Thus far we have demonstrated that the TLR-4 ligand LPS was able to induce NF-κB binding at two sites on the proximal MIG promoter. In order to determine the functional significance of LPS-induced NF-κB proteins binding to the − 154 and − 129 sites of the MIG promoter, we used transient transfection assays. We designed promoter constructs containing the wild type − 284 to + 43 fragment of the MIG promoter upstream of a firefly luciferase reporter gene (p284) or constructs containing mutations in the − 154 (pM154), − 129 (pM129) and both − 154 and − 129 (pM129/154) NF-κB-like sites identical to the mutant probes used in the EMSA studies. Mutations at the individual NF-κB-like sites did not inhibit the synergy between LPS and IFN-γ but mutations at both the − 154 and − 129 sites completely eliminated the synergy (Fig. 7). Additionally, transfection assays using a construct containing four copies of the γ-responsive element-1 (γRE-1) alone upstream of a firefly luciferase reporter also failed to demonstrate the synergy induced by LPS and IFN-γ indicating that the γRE-1 is not sufficient to mediate the synergy (data not shown).

Surprisingly mutations at the − 154 and − 129 sites (individually and combined) decreased the induction of MIG expression by IFN-γ alone. There was a significant inhibition of IFN-γ induced MIG between the wild type promoter construct p284 and the mutants: pM154 ($p = 0.0157$), pM129 ($p = 0.003$), and pM129/154 ($p < 0.0001$). The inhibition was greatest in the dual − 154 and − 129 mutated construct, but was still significant in the − 129 mutant and to a lesser degree in the − 154 mutant. Furthermore, the construct containing four copies of the γRE-1 alone, with no NF-κB-like sites, also demonstrated decreased response to IFN-γ stimulation (data not shown). These transfection studies support the notion that NF-κB binding at both the − 154 and − 129 sites is necessary for the synergistic induction of MIG by LPS and IFN-γ. Furthermore, they indicate that these *cis* elements may play a role in the maximal induction of MIG by IFN-γ alone.

### NF-κB p50 Helps Mediate the Synergy between LPS and IFN-γ on MIG Expression in Macrophages

The inhibitor, EMSA and transfection data in Figs. 5–7 suggest that the − 154 and − 129 NF-κB binding sites are necessary for both the synergy between LPS and IFN-γ on MIG expression as well as the induction of MIG by IFN-γ alone. Thus, we wanted to evaluate the role of NF-κB p50 proteins in mediating the synergy between LPS and IFN-γ. To do this, Northern analysis was performed on total RNA from thioglycollate elicited peritoneal macrophages from NF-κB p50 null mice stimulated with varying doses of LPS +/− IFN-γ. As seen in Fig. 8, there was a shift in the dose response curve for induction of MIG mRNA by LPS and IFN-γ. Low doses of IFN-γ did not

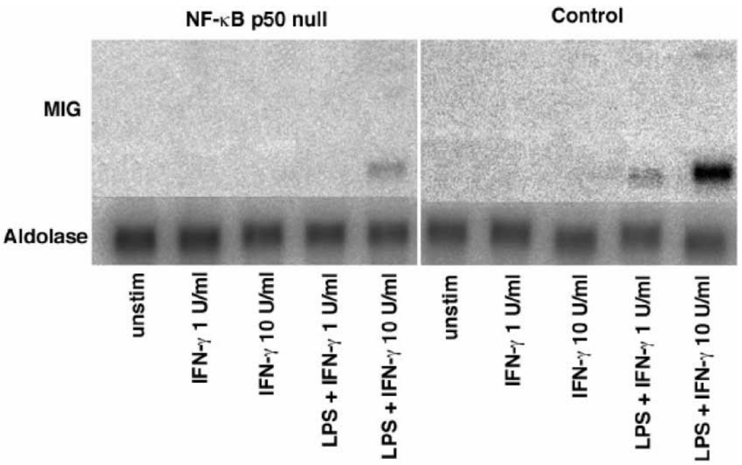

FIGURE 8 NF-κB p50 is required for the synergistic induction of MIG by LPS and IFN-γ.mRNA was isolated and Northern analysis was performed on thioglycollate elicited peritoneal macrophages from NF-κB p50 null or control mice stimulated with LPS (0.1 ng/ml) +/− IFN γ (1 or 10 U/ml) for 6 h. This is a representative blot of three identical experiments.

induce MIG mRNA in either the control or NF-κB null mice, but 10 U of IFN-γ + 0.1 ng/ml of LPS markedly induced MIG in control mice where as it had a minimal effect on cells from the NF-κB p50 null mice. In fact, faint induction of MIG was present in control mice with as little as 1 U of IFN-γ + 0.1 ng/ml of LPS (Fig. 8). These data suggest that the NF-κB p50 member plays a critical (non-redundant) role in both IFN-γ-induced and IFN-γ + LPS-induced MIG expression.

### DISCUSSION

MIG was originally described in both humans and mice as a gene specifically induced by IFN-γ (Farber, 1990,1992, 1993). By binding to its receptor CXCR3, MIG attracts both CD4+ and CD8+ T cells and has been implicated in contributing to the host inflammatory response against infection and tumor immunity (Liao *et al.*, 1995; Strieter *et al.*, 1995). It is also involved in the pathogenesis of a number of autoimmune disorders as well as the development of atherosclerosis (Liu *et al.*, 2001; Belperio *et al.*, 2002; Mahad *et al.*, 2002; Yun *et al.*, 2002). Recently, by examining MIG null mice, Park *et al.*, have revealed a role for MIG in skewing toward a TH1 immune response as well as contributing to the optimal generation of humoral immune responses to intracellular bacterial infections (Park *et al.*, 2002). In addition, this group has shown that IFN-γ can induce MIG in dendritic cells (Park *et al.*, 2002). Taken together, these data implicate MIG as playing a key role in the adaptive immune response by recruiting T cells and enhancing their interactions with B cells and dendritic cells (Park *et al.*, 2002).

Our experiments sought to dissect the transcriptional mechanisms by which signals from pathogens and the host cooperate to enhance the immune response. The data clearly demonstrate the ability of bacterial, viral and mycotic products to enhance MIG expression. In particular, while low doses of IFN-γ fail to induce MIG, the addition of these infectious products results in substantial MIG production. At the molecular level, the integration of cellular signals (IFN-γ) and infectious signals (TLR ligation) was mediated by STAT1α and NF-κB, respectively. Pharmacologic inhibition of NF-κB activation inhibited this synergy and the synergy was markedly decreased in macrophages derived from p50 KO mice. Additionally, by mutating the proximal MIG promoter, we demonstrated a key role for the −154 and −129 NF-κB binding sites in facilitating the ability of LPS to enhance IFN-γ-induced MIG expression. Furthermore, the transfection studies identified the −154 and −129 sites were important to the induction of MIG by IFN-γ alone (Fig. 7).

In this report we demonstrate that the maximal production of the T cell attractant MIG by macrophages is the result of the collaboration between Toll-like receptor engagement and IFN-γ. Although it has been previously shown that TNF-α can synergize with IFN-γ to promote MIG production by fibroblasts, the synergy induced by TLR-ligation on macrophages is direct and independent of TNF-α. Given that the ligands for the TLRs are byproducts of infectious agents such as bacteria, viruses and fungi, these observations define another mechanism whereby the innate immune response serves to promote adaptive immunity. The inability of TLR engagement alone to induce MIG suggests that in the absence of IFN-γ (presumably initially produced by NK cells) the recruitment of T cells by MIG to the site of infection does not occur. Means *et al.*, noted that the TLR 5 ligand bacterial flagellin could only induce MIG production from dendritic cells in conjunction with autocrine IFN-β (Means *et al.*, 2003). Our data are different in that in both the macrophage cell line and the primary macrophages, TLR 2, 3, 4 and 9 engagement alone did not induce MIG. Despite the fact that CpG is known to cause the induction of IFN-β in macrophages, IFN-β could not substitute for IFN-γ in terms of the synergy (Means *et al.*, 2003). Thus, perhaps the role of MIG in promoting autoimmunity is due in part to the dysregulation of this two signal requirement. For example, a genetic predisposition to loosely regulate IFN-γ may result in the inappropriate induction of MIG in the setting of TLR ligation and thus the recruitment of T cells to target organs.

The molecular pathways described here also have potential clinical implications. First, MIG has been implicated in playing a role in a number of autoimmune disorders (Belperio *et al.*, 2002; Mahad *et al.*, 2002; Yun *et al.*, 2002). The critical role of NF-κB in the production of maximal MIG suggests that pharmacologic inhibition of NF-κB activation may not only lead to a decrease in inflammation in general but also may serve to decrease MIG mediated recruitment to target organs. In this regard, NF-κB inhibitors such as PS-341 (Velcade) have already been approved for human use (Twombly, 2003). It will be interesting to determine if in certain animal models of autoimmunity such inhibitors have the ability to decrease MIG expression. Alternatively, MIG has also been implicated in the anti-tumor immune response (Ruehlmann *et al.*, 2001). Thus, as many of the TLR agonists such CpG are being pursued as adjuvants in immunotherapy, it is possible that their effects are in part through the up-regulation of MIG.

### *Acknowledgements*

The authors would like to thank David B. Jacoby, MD and Sekhar Reddy, PhD for their critical reading of the manuscript.

This work was supported by grants from the National Institutes of Health (K08 HL03993) and the American Lung Association (RG-061-N) (MRH) and NIH, 1R01CA098109-01 and D.O.D. BC021222 (JDP).

### *References*

Amichay, D., Gazzinelli, R., Karupiah, G., Moench, T., Sher, A. and Farber, J. (1996) "Genes for chemokine MuMig and crg-2 are induced in protozoan and viral infections in response to IFN-γ with patterns of tissue expression that suggest nonredundant roles *in vivo*", *J. Immol.* **157**, 4511–4520.

Belperio, J.A., Keane, M.P., Burdick, M.D., *et al.* (2002) "Critical role for CXCR3 chemokine biology in the pathogenesis of bronchiolitis obliterans syndrome", *J. Immunol.* **169**, 1037–1049.

Dunne, A. and O'Neill, L.A. (2003) "The interleukin-1 receptor/Toll-like receptor superfamily: signal transduction during inflammation and host defense", *Sci. STKE* **2003**, re3.

Farber, J.M. (1990) "A macrophage mRNA selectively induced by γ-interferon encodes a member of the platelet factof 4 family of cytokines", *Proc. Natl Acad. Sci. USA* **87**, 5238–5242.

Farber, J.M. (1992) "A collection of mRNA species that are inducible in the RAW 264.7 mouse macrophage cell line by gamma interferon and other agents", *Mol. Cell Biol.* **12**, 1535–1545.

Farber, J.M. (1993) "HuMIG: a new member of the chemokine family of cytokines", *Biochem. Biophys. Res. Commun.* **192**, 223–230.

Gasperini, S., Marchi, M., Calzetti, F., *et al.* (1999) "Gene expression and production of the monokine induced by IFN-gamma (MIG), IFN-inducible T cell alpha chemoattractant (I-TAC), and IFN-gamma-inducible protein-10 (IP-10) chemokines by human neutrophils", *J. Immunol.* **162**, 4928–4937.

Hiroi, M. and Ohmori, Y. (2003) "The transcriptional coactivator CREB-binding protein cooperates with STAT1 and NF-kappa B for synergistic transcriptional activation of the CXC ligand 9/monokine induced by interferon-gamma gene", *J. Biol. Chem.* **278**, 651–660.

Horton, M.R., McKee, C.M., Bao, C., *et al.* (1998) "Hyaluronan fragments synergize with interferon-gamma to induce the C-X-C chemokines mig and interferon-inducible protein-10 in mouse macrophages", *J. Biol. Chem.* **52**, 35088–35094.

Horton, M.R., Boodoo, S. and Powell, J.D. (2002) "NF-kappa B activation mediates the cross-talk between extracellular matrix and interferon-gamma (IFN-gamma) leading to enhanced monokine induced by IFN-gamma (MIG) expression in macrophages", *J. Biol. Chem.* **277**, 43757–43762.

Jones, B.W., Means, T.K., Heldwein, K.A., *et al.* (2001) "Different Toll-like receptor agonists induce distinct macrophage responses", *J. Leukoc. Biol.* **69**, 1036–1044.

Liao, F., Rabin, R.L., Yannelli, J.R., Koniaris, L.G., Vanguri, P. and Farber, J.M. (1995) "Human Mig chemokine: biochemical and functional characterization", *J. Exp. Med.* **182**, 1301–1314.

Liu, M.T., Armstrong, D., Hamilton, T.A. and Lane, T.E. (2001) "Expression of Mig (monokine induced by interferon-gamma) is important in T lymphocyte recruitment and host defense following viral infection of the central nervous system", *J. Immunol.* **166**, 1790–1795.

Loetscher, M., Gerber, B., Leotscher, P., *et al.* (1996) "Chemokine receptor specific for IP-10 and Mig: structure, function, and expression in activated T-lymphocytes", *J. Exp. Med.* **184**, 963–969.

Luster, A.D., Unkeleee, J.C. and Ravetch, J.V. (1985) "γ-Interferon transcription regulates an early-response gene containing homolgy to platlet proteins", *Nature* **315**, 672–676.

Maeyer, E. and Maeyer-Guignard, J.D.E. (1992) "Interferon-gamma", *Curr. Opin. Immunol.* **4**, 321.

Mahad, D.J., Howell, S.J. and Woodroofe, M.N. (2002) "Expression of chemokines in the CSF and correlation with clinical disease activity in patients with multiple sclerosis", *J. Neurol. Neurosurg. Psychiatry* **72**, 498–502.

Marino, M., Dunn, A., Grail, D., *et al.* (1997) "Characterization of tumor necrosis factor-defecient mice", *Proc. Natl Acad. Sci. USA* **94**, 8093–8098.

Mbawuike, I. and Herscowitz, H. (1989) "MH-S, a murine alveolar macrophage cell line: morphological, cytochemical, and functional characteristics", *J. Leuk. Biol.* **46**, 119–127.

Means, T.K., Hayashi, F., Smith, K.D., Aderem, A. and Luster, A.D. (2003) "The Toll-like receptor 5 stimulus bacterial flagellin induces maturation and chemokine production in human dendritic cells", *J. Immunol.* **170**, 5165–5175.

Nathan, C. (1987) "Secretory products of macrophages", *J. Clin. Invest.* **79**, 319–322.

Ohmori, Y. and Hamilton, T. (1993) "Cooperative interaction between interferon stimulus response element and kappa B sequence motifs controls IFN gamma- and lipopolysaccharide-stimulated transcription from the murine IP-10 promoter", *J. Biol. Chem.* **268**, 6677–6688.

Ohmori, Y. and Hamliton, T. (1995) "The interferon-stimulated response element and a kappa B site mediate synergistic induction of murine IP-10 gene transcription by IFN-gamma and TNF-alpha", *J. Immunol.* **154**, 5235–5244.

Ohmori, Y., Schreiber, R. and Hamilton, T. (1997) "Synergy between interferon-γ and tumor necrosis factor-α in transcriptional activation is mediated by cooperation between signal transducer and activator of transcription 1 and nuclear factor κB", *J. Biol. Chem.* **272**, 14899–14907.

Park, M.K., Amichay, D., Love, P., *et al.* (2002) "The CXC chemokine murine monokine induced by IFN-gamma (CXC chemokine ligand 9) is made by APCs, targets lymphocytes including activated B cells, and supports antibody responses to a bacterial pathogen *in vivo*", *J. Immunol.* **169**, 1433–1443.

Powell, J.D., Lerner, C.G., Ewoldt, G.R. and Schwartz, R.H. (1999) "The -180 site of the IL-2 promoter is the target of CREB/CREM binding in T cell anergy", *J Immunol.* **163**, 6631–6639.

Ruehlmann, J.M., Xiang, R., Niethammer, A.G., *et al.* (2001) “MIG (CXCL9) chemokine gene therapy combines with antibody-cytokine fusion protein to suppress growth and dissemination of murine colon carcinoma”, *Cancer Res.* **61**, 8498–8503.

Strieter, R.M., Polverini, P.J., Kunkel, S.L., *et al.* (1995a) “The functional role of the ELR motif in CXC chemokine-mediated angiogenesis”, *J. Biol. Chem.* **270**, 27348–27357.

Strieter, R.M., Polverini, P.J., Arenberg, D.A. and Kunkel, S.L. (1995b) “The role of CXC chemokines as regulators of angiogenesis”, *Shock* **4**, 155–160.

Sun, H., Kundu, N., Dorsey, R., Jackson, M.J. and Fulton, A.M. (2001) “Expression of the chemokines IP-10 and Mig in IL-10 transduced tumors”, *J. Immunother.* **24**, 138–143.

Takeuchi, O. and Akira, S. (2001) “Toll-like receptors; their physiological role and signal transduction system”, *Int. Immunopharmacol.* **1**, 625–635.

Twombly, R. (2003) “First proteasome inhibitor approved for multiple myeloma”, *J Natl Cancer Inst.* **95**, 845.

Yun, J.J., Fischbein, M.P., Whiting, D., *et al.* (2002) “The role of MIG/CXCL9 in cardiac allograft vasculopathy”, *Am. J. Pathol.* **161**, 1307–1313.

